# IMGT, the international ImMunoGeneTics information system^®^: a standardized approach for immunogenetics and immunoinformatics

**DOI:** 10.1186/1745-7580-1-3

**Published:** 2005-09-20

**Authors:** Marie-Paule Lefranc

**Affiliations:** 1 IMGT, the international ImMunoGeneTics information system^®^, Université Montpellier II, Institut Universitaire de France, Laboratoire d'ImmunoGénétique Moléculaire LIGM, UPR CNRS 1142, Institut de Génétique Humaine IGH, 141 rue de la Cardonille, 34396 Montpellier Cedex 5, France

**Keywords:** IMGT, antibody, immunoglobulin, T cell receptor, superfamily, MHC, HLA, ontology, database, information system, knowledge resource, immunoinformatics, immunogenetics, Collier de Perles, three-dimensional, 3D structure, polymorphism, annotation

## Abstract

IMGT, the international ImMunoGeneTics information system^®^, was created in 1989 by the Laboratoire d'ImmunoGénétique Moléculaire (LIGM) (Université Montpellier II and CNRS) at Montpellier, France. IMGT is a high quality integrated knowledge resource specialized in immunoglobulins (IG), T cell receptors (TR), major histocompatibility complex (MHC) of human and other vertebrates, and related proteins of the immune system (RPI) of any species which belong to the immunoglobulin superfamily (IgSF) and to the MHC superfamily (MhcSF). IMGT consists of five databases, ten on-line tools and more than 8,000 HTML pages of Web resources. IMGT provides a common access to standardized data from genome, genetics, proteome and three-dimensional structures. The accuracy and the consistency of IMGT data are based on IMGT-ONTOLOGY, a semantic specification of terms to be used in immunogenetics and immunoinformatics. IMGT-ONTOLOGY comprises six main concepts: IDENTIFICATION, CLASSIFICATION, DESCRIPTION, NUMEROTATION, ORIENTATION and OBTENTION. Based on these concepts, the controlled vocabulary and the annotation rules necessary for the immunogenetics data identification, classification, description and numbering and for the management of IMGT knowledge are defined in the IMGT Scientific chart. IMGT is the international reference in immunogenetics and immunoinformatics for medical research (repertoire analysis of the IG antibody sites and of the TR recognition sites in autoimmune and infectious diseases, AIDS, leukemias, lymphomas, myelomas), veterinary research (IG and TR repertoires in farm and wild life species), genome diversity and genome evolution studies of the adaptive immune responses, biotechnology related to antibody engineering (single chain Fragment variable (scFv), phage displays, combinatorial libraries, chimeric, humanized and human antibodies), diagnostics (detection and follow up of residual diseases) and therapeutical approaches (grafts, immunotherapy, vaccinology). IMGT is freely available at .

## Introduction

IMGT, the international ImMunoGeneTics information system^®^[1,2], was created in 1989, by Marie-Paule Lefranc, at the Laboratoire d'ImmunoGénétique Moléculaire (LIGM) (Université Montpellier II and CNRS) at Montpellier, France, in order to standardize and manage the complexity of the immunogenetics data. Fifteen years later, IMGT is the international reference in immunogenetics and immunoinformatics, and provides a high quality integrated knowledge resource, specialized in the immunoglobulins (IG) and T cell receptors (TR), major histocompatibility complex (MHC) of human and other vertebrates, and related proteins of the immune systems (RPI) of any species which belong to the immunoglobulin superfamily (IgSF) and to the MHC superfamily (MhcSF)[3-13]. The number of potential protein forms of the antigen receptors, IG and TR, is almost unlimited. The potential repertoire of each individual is estimated to comprise about 10^12 ^different IG (or antibodies) and TR, and the limiting factor is only the number of B and T cells that an organism is genetically programmed to produce. This huge diversity is inherent to the particularly complex and unique molecular synthesis and genetics of the antigen receptor chains. This includes biological mechanisms such as DNA molecular rearrangements in multiple loci (three for IG and four for TR in humans) located on different chromosomes (four in humans), nucleotide deletions and insertions at the rearrangement junctions (or N-diversity), and somatic hypermutations in the IG loci (see FactsBooks[3,4] for review). Although IMGT was initially implemented for the IG, TR and MHC of human and other vertebrates [6], data and knowledge management standardization, based on the IMGT unique numbering [14-19], has now been extended to the IgSF [15-17,20-22] and MhcSF [18,23,24] of any species. Thus, standardization in IMGT contributed to data enhancement of the system and new expertised data concepts were readily incorporated.

**Figure 1:**
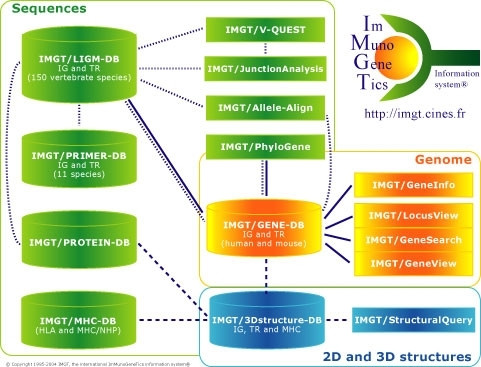
IMGT, the international ImMunoGeneTics information system^® ^. Databases and tools for sequences, genes and structures are in green, yellow and blue, respectively. The IMGT Repertoire and other Web resources are not shown. Interactions in the genetics, genomics and structural approaches are represented with dotted, continuous and broken lines, respectively.

IMGT, the international ImMunoGeneTics information system^®^ consists of five databases, ten on-line tools and Web resources [1,2]. Databases include sequence databases (IMGT/LIGM-DB, IMGT/PRIMER-DB and IMGT/MHC-DB), one genome database (IMGT/GENE-DB) and one three-dimensional (3D) structure database (IMGT/3Dstructure-DB) [1,2] (Figure 1). Interactive tools are provided for sequence analysis (IMGT/V-QUEST, IMGT/JunctionAnalysis, IMGT/Allele-Align, IMGT/PhyloGene), genome analysis (IMGT/LocusView, IMGT/GeneView, IMGT/GeneSearch, IMGT/CloneSearch and IMGT/GeneInfo) and 3D structure analysis (IMGT/StructuralQuery) [1,2] (Figure 1). Web resources ("IMGT Marie-Paule page") comprise more than 8,000 HTML pages of synthesis [IMGT Repertoire (for IG and TR, MHC, RPI)], knowledge [IMGT Scientific chart, IMGT Education (IMGT Lexique, Aide-mémoire, Tutorials, Questions and answers), IMGT Medical page, IMGT Veterinary page, IMGT Biotechnology page, IMGT Index], and external links [IMGT Immunoinformatics page, IMGT Bloc-notes (Interesting links, etc.) and IMGT other accesses (SRS, BLAST, etc.)] [2]. Despite the heterogeneity of these different components, all data in the IMGT information system are expertly annotated. The accuracy, the consistency and the integration of the IMGT data, as well as the coherence between the different IMGT components (databases, tools and Web resources) are based on IMGT-ONTOLOGY[5], which provides a semantic specification of the terms to be used in immunogenetics and immunoinformatics. IMGT-ONTOLOGY, the first ontology in the domain, has allowed the management of knowledge in immunogenetics [2,25] and provided standardization for immunogenetics data from genome, genetics, proteome and 3D structures [3-13]. IMGT-ONTOLOGY concepts are available, for the biologists and IMGT users, in the IMGT Scientific chart[2], and for the computing scientists, in IMGT-ML which uses XML (eXtensible Markup Language) Schema [26].

### IMGT-ONTOLOGY concepts and IMGT Scientific chart rules

The IMGT Scientific chart[2] comprises the controlled vocabulary and the annotation rules necessary for the immunogenetics data identification, description, classification and numbering and for knowledge management in the IMGT information system. Standardized keywords, labels and annotation rules, standardized IG and TR gene nomenclature, the IMGT unique numbering, and standardized origin/methodology were defined, respectively, based on the six main concepts of IMGT-ONTOLOGY: IDENTIFICATION, CLASSIFICATION, DESCRIPTION, NUMEROTATION, ORIENTATION and OBTENTION[2,5] (Table 1). The IMGT Scientific chart is available as a section of the IMGT Web resources (IMGT Marie-Paule page). Examples of IMGT expertised data concepts derived from the IMGT Scientific chart rules are shown in Table 1.

**Table 1:** IMGT-ONTOLOGY concepts, IMGT Scientific chart rules and examples of IMGT expertised data concepts.

IMGT-ONTOLOGY main concepts 5	IMGT Scientific chart rules [2]	Examples of IMGT expertised data concepts [2]
IDENTIFICATION	Standardized keywords [5]	Species, molecule type, receptor type, chain type, gene type, structure, functionality, specificity
CLASSIFICATION	Reference sequences Standardized IG and TR gene nomenclature (group, subgroup, gene, allele) [5]	Nomenclature of the human IG and TR genes (entry in 1999 in GDB, HGNC [27] and LocusLink at NCBI) [3, 4] Alignment of alleles [3, 4] Nomenclature of the IG and TR genes of all vertebrate species
DESCRIPTION	Standardized labels and annotations [5]	Core (V-, D-, J-, C-REGION) Prototypes [5] Labels for sequences Labels for 2D and 3D structures
NUMEROTATION	IMGT unique numbering [14-18] for: V- and V-LIKE-DOMAINs [16] C- and C-LIKE-DOMAINs [17] G- and G-LIKE-DOMAINs [18]	Protein displays IMGT Colliers de Perles [19] FR-IMGT and CDR-IMGT delimitations [16] Structural loops and beta strands delimitations [16, 17]
ORIENTATION	Orientation of genomic instances relative to each other	Chromosome orientation Locus orientation Gene orientation DNA strand orientation
OBTENTION	Standardized origin Standardized methodology [2]	

The IMGT Scientific chart rules, based on the IMGT-ONTOLOGY concepts [5], are used in the three major IMGT biological approaches, genomics, genetics and structural approaches [2], and corresponding data (Genes, Sequences, 3D structures) are available in the IMGT components (databases, tools and Web resources) [1,7-13].

### IMGT sequence databases, tools and Web resources

IMGT sequence databases, tools and Web resources correspond to the IMGT genetics approach that refers to the study of genes in relation with their polymorphisms, mutations, expression, specificity and evolution (Table 2). The IMGT sequence knowledge management and the IMGT genetics approach heavily rely on the DESCRIPTION concept (and particularly on the V-REGION, D-REGION, J-REGION and C-REGION core concepts for the IG and TR), on the CLASSIFICATION concept (gene and allele concepts) and on the NUMEROTATION concept (IMGT unique numbering [14-18]).

**Table 2:** The IMGT sequence databases, sequence analysis tools and Web resources

IMGT sequence databases [1]	IMGT sequence analysis tools [1]	IMGT Repertoire"Proteins and alleles" section [2](2)
IMGT/LIGM-DB [7] IMGT/PRIMER-DB [1] IMGT/MHC-DB [28]	IMGT/V-QUEST [10] IMGT/JunctionAnalysis [11] IMGT/Allele-Align IMGT/PhyloGene [12] IMGT/Automat [29, 30] (1)	Alignments of alleles IG and TR [3, 4] Alignments of alleles RPI [22] Protein displays IG and TR [3, 4, 16, 17] Protein displays MHC [18] Protein displays RPI [16-18, 21] Tables of alleles IG and TR Tables of alleles RPI [22, 24] Allotypes Isotypes, etc.

(1) IMGT/Automat [29, 30] is an integrated internal IMGT Java tool which automatically performs the annotation of rearranged cDNA sequences that represent the half of the IMGT/LIGM-DB content. So far 7,418 human and mouse IG and TR cDNA sequences have been automatically annotated by the IMGT/Automat tool, with annotations being as reliable and accurate as those provided by a human annotator.

(2) IMGT publications from the IMGT Repertoire "Proteins and alleles" section are available as pdf in IMGT Locus in Focus , in IMGT Index (see also [2]).

#### IMGT sequence databases

##### IMGT/LIGM-DB

IMGT/LIGM-DB is the comprehensive IMGT database of IG and TR nucleotide sequences from human and other vertebrate species, with translation for fully annotated sequences [7]. It was created in 1989 by LIGM (Montpellier, France), and is on the Web since July 1995 [6]. In August 2005, IMGT/LIGM-DB contained more than 96,500 sequences of 150 vertebrate species [7]. The unique source of data for IMGT/LIGM-DB is EMBL, which shares data with the other two generalist databases GenBank and DNA DataBank of Japan (DDBJ). Based on expert analysis, specific detailed annotations are added to IMGT flat files. The annotation procedure includes the IDENTIFICATION of the sequences, the CLASSIFICATION of the IG and TR genes and alleles, and the DESCRIPTION of all IG and TR specific and constitutive motifs within the nucleotide sequences. The Web interface allows searches according to immunogenetic specific criteria and is easy to use without any knowledge in a computing language. Selection is displayed at the top of the resulting sequences pages, so the users can check their own queries. Users have the possibility to modify their request or consult the results with a choice of nine possibilities. The IMGT/LIGM-DB annotations (gene and allele name assignment, labels) allow data retrieval not only from IMGT/LIGM-DB, but also from other IMGT databases. Thus, the IMGT/LIGM-DB accession numbers of the cDNA expressed sequences for each human and mouse IG and TR gene are available, with direct links to IMGT/LIGM-DB, in the IMGT/GENE-DB entries. IMGT/LIGM-DB data are also distributed by anonymous FTP servers at CINES  and EBI  and from many Sequence Retrieval System (SRS) sites . IMGT/LIGM-DB can be searched by BLAST or FASTA on different servers (EBI, IGH, INFOBIOGEN, Institut Pasteur, etc.).

##### IMGT/PRIMER-DB

IMGT/PRIMER-DB[1] is the IMGT oligonucleotide primer database for IG and TR, created by LIGM, Montpellier in collaboration with EUROGENTEC S.A., Belgium, on the Web since February 2002. In August 2005, IMGT/PRIMER-DB contained 1,827 entries. IMGT/PRIMER-DB provides standardized information on oligonucleotides (or Primers) and combinations of primers (Sets, Couples) for IG and TR. These primers are useful for combinatorial library constructions, scFv, phage display or microarray technologies. The IMGT Primer cards are linked to the IMGT/LIGM-DB flat files, IMGT Colliers de Perles and IMGT Alignments of alleles (IMGT Repertoire) of the IMGT/LIGM-DB reference sequence used for the primer description.

##### IMGT/MHC-DB

IMGT/MHC-DB[28] comprises databases hosted at EBI and includes a database of human MHC allele sequences or IMGT/MHC-HLA, developed by Cancer Research UK and maintained by ANRI, London, UK, on the Web since December 1998, and a database of MHC sequences from non human primates IMGT/MHC-NHP, curated by BPRC, The Netherlands, on the Web since April 2002.

#### IMGT sequence analysis tools

The IMGT sequence analysis tools comprise IMGT/V-QUEST[10], for the identification of the V, D and J genes and of their mutations, IMGT/JunctionAnalysis[11] for the analysis of the V-J and V-D-J junctions which confer the antigen receptor specificity, IMGT/Allele-Align for the detection of polymorphisms, and IMGT/PhyloGene[12] for gene evolution analyses.

##### IMGT/V-QUEST

IMGT/V-QUEST (V-QUEry and STandardization) is an integrated software for IG and TR [10]. This tool, easy to use, analyses an input IG or TR germline or rearranged variable nucleotide sequence. The IMGT/V-QUEST results comprise the identification of the V, D and J genes and alleles and the nucleotide alignments by comparison with sequences from the IMGT reference directory, the FR-IMGT and CDR-IMGT delimitations based on the IMGT unique numbering, the translation of the input sequence, the display of nucleotide and amino acid mutations compared to the closest IMGT reference sequence, the identification of the JUNCTION and results from IMGT/JunctionAnalysis (default option), and the two-dimensional (2D) IMGT Collier de Perles representation of the V-REGION [10] ("IMGT/V-QUEST output" in IMGT/V-QUEST Documentation).

##### IMGT/JunctionAnalysis

IMGT/JunctionAnalysis[11] is a tool, complementary to IMGT/V-QUEST, which provides a thorough analysis of the V-J and V-D-J junction of IG and TR rearranged genes. IMGT/JunctionAnalysis identifies the D-GENEs and alleles involved in the IGH, TRB and TRD V-D-J rearrangements by comparison with the IMGT reference directory, and delimits precisely the P, N and D regions [11] ("IMGT/JunctionAnalysis output results" in IMGT/JunctionAnalysis Documentation). Several hundreds of junction sequences can be analysed simultaneously.

##### IMGT/Allele-Align

IMGT/Allele-Align is used for the detection of polymorphisms. It allows the comparison of two alleles highlighting the nucleotide and amino acid differences.

##### IMGT/PhyloGene

IMGT/PhyloGene[12] is an easy to use tool for phylogenetic analysis of variable region (V-REGION) and constant domain (C-DOMAIN) sequences. This tool is particularly useful in developmental and comparative immunology. The users can analyse their own sequences by comparing with the IMGT standardized reference sequences for human and mouse IG and TR [12] (IMGT/PhyloGene Documentation).

#### IMGT sequence Web resources

The IMGT sequence Web resources are compiled in the IMGT Repertoire "Proteins and alleles" section that include Alignments of alleles, Proteins displays, Tables of alleles, Allotypes, Isotypes, etc. (Table 2). Standardized IMGT criteria for amino acid sequence analysis are described in [31].

### IMGT gene databases, tools and Web resources

IMGT gene databases, tools and Web resources correspond to the IMGT genomics approach that refers to the studies of the genes within their loci and on their chromosome [2] (Table 3).

**Table 3:** The IMGT gene database, genome analysis tools and Web resources

IMGT genome database [1]	IMGT genome analysis tools [1]	IMGT Repertoire"Locus and genes" section [2] (1)
IMGT/GENE-DB [8]	IMGT/LocusView IMGT/GeneView IMGT/GeneSearch IMGT/CloneSearch IMGT/GeneInfo [13]	Chromosomal localizations [3, 4] Locus representations [3, 4] Locus description Gene exon/intron organization Gene exon/intron splicing sites Gene tables Potential germline repertoires Lists of genes Correspondence between nomenclatures [3, 4]

(1) IMGT Web resources (IMGT Marie-Paule page) also include IMGT Index, IMGT Education (IMGT Lexique, Aide-mémoire, Tutorials, Questions and answers), The IMGT Medical page, The IMGT Veterinary page, The IMGT Biotechnology page, The IMGT Immunoinformatics page, IMGT Bloc-notes (Interesting links, etc.) [2] which are not detailed in this paper.

(2) IMGT publications from the IMGT Repertoire "Locus and genes" section are available as pdf in IMGT Locus in Focus , in IMGT Index (see also [2]).

#### IMGT/GENE-DB, the IMGT gene database

Genomic data are managed in IMGT/GENE-DB, which is the comprehensive IMGT genome database [8]. IMGT/GENE-DB, created by LIGM (Montpellier, France) is on the Web since January 2003. In August 2005, IMGT/GENE-DB contained 1,377 genes and 2,207 alleles (673 IG and TR genes and 1,209 alleles from *Homo sapiens*, and 704 IG and TR genes and 998 alleles from *Mus musculus*, *Mus cookii*, *Mus pahari*, *Mus spretus*, *Mus saxicola*, *Mus minutoïdes*). All the human and mouse IG and TR genes are available in IMGT/GENE-DB. Based on the IMGT CLASSIFICATION concept, all the human IMGT gene names [3,4] were approved by the Human Genome Organisation (HUGO) Nomenclature Committee HGNC in 1999 [27], and entered in IMGT/GENE-DB [8], Genome DataBase GDB (Canada) [32], LocusLink and Entrez Gene at NCBI (USA) [33], and GeneCards [34]. Reciprocal links exist between IMGT/GENE-DB, and the generalist nomenclature (HGNC Genew) and genome databases (GDB, LocusLink and Entrez at NCBI, and GeneCards). All the mouse IG and TR gene names with IMGT reference sequences were provided by IMGT to HGNC and to the Mouse Genome Database (MGD) [35] in July 2002. Queries in IMGT/GENE-DB can be performed according to IG and TR gene classification criteria and IMGT reference sequences have been defined for each allele of each gene based on one or, whenever possible, several of the following criteria: germline sequence, first sequence published, longest sequence, mapped sequence [2]. IMGT/GENE-DB interacts dynamically with IMGT/LIGM-DB [7] to download and display gene-related sequence data. As an example ans as mentioned earlier, the IMGT/GENE-DB entries provide the IMGT/LIGM-DB accession numbers of the IG and TR cDNA sequences which contain a given V, D, J or C gene. This is the first example of an interaction between IMGT databases using the CLASSIFICATION concept.

#### IMGT gene analysis tools

The IMGT gene analysis tools comprise IMGT/LocusView, IMGT/GeneView, IMGT/GeneSearch, IMGT/CloneSearch and IMGT/GeneInfo. IMGT/LocusView and IMGT/GeneView manage the locus organization and the gene location and provide the display of physical maps for the human IG, TR and MHC loci and for the mouse TRA/TRD locus. IMGT/LocusView allows to view genes in a locus and to zoom on a given area. IMGT/GeneView allows to view a given gene in a locus. IMGT/GeneSearch allows to search for genes in a locus based on IMGT gene names, functionality or localization on the chromosome. IMGT/CloneSearch provides information on the clones that were used to build the locus contigs displayed in IMGT/LocusView (accession numbers are from IMGT/LIGM-DB, gene names from IMGT/GENE-DB, and clone position and orientation, and overlapping clones from IMGT/LocusView). IMGT/GeneInfo[13] provides and displays information on the potential TR rearrangements in human and mouse.

#### IMGT gene Web resources

The IMGT gene Web resources are compiled in the IMGT Repertoire "Locus and genes" section that includes Chromosomal localizations, Locus representations, Locus description, Gene exon/intron organization, Gene exon/intron splicing sites, Gene tables, Potential germline repertoires, the complete lists of human and mouse IG and TR genes, and the correspondences between nomenclatures [3,4] (Table 3). The IMGT Repertoire "Probes and RFLP" section provides additional data on gene insertion/deletion.

### IMGT structure database, tool and Web resources

The IMGT structural approach refers to the study of the 2D and 3D structures of the IG, TR, MHC and RPI, and to the antigen or ligand binding characteristics in relation with the protein functions, polymorphisms and evolution (Table 4). The structural approach relies on the CLASSIFICATION concept (IMGT gene and allele names), DESCRIPTION concept (receptor and chain description, domain delimitations), and NUMEROTATION concept (amino acid positions according to the IMGT unique numbering [14-18]).

**Table 4:** IMGT structure database, analysis tool and Web resources

IMGT structural database [1]	IMGT structural analysis tool [1]	IMGT Repertoire"2D and 3D structures" section [2]
IMGT/3D structure-DB [15]	IMGT/StructuralQuery [15]	2D Colliers de Perles IG and TR [3, 4, 16, 17, 19] (1) 2D Colliers de Perles MHC [18, 36] 2D Colliers de Perles RPI [16-18, 21, 22, 24, 37] IMGT classes for amino acid characteristics [31] IMGT Colliers de Perles reference profiles [31] 3D representations (1)

(1) Cover of the Nucleic Acids Research 1999 database issue

**Figure 2:**
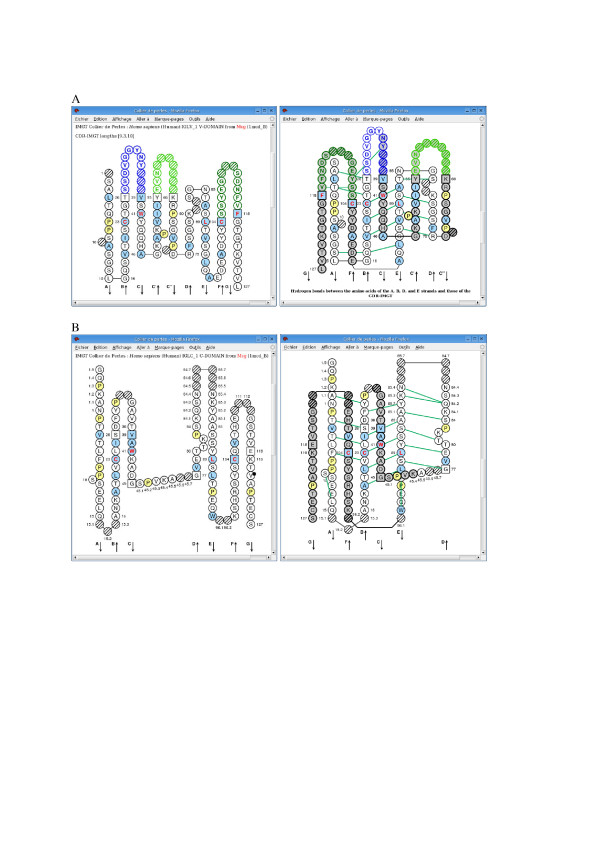
IMGT Colliers de Perles of a V-DOMAIN (A) and of a C-DOMAIN (B) (code PDB 1mcd in IMGT/3Dstructure-DB [9]). IMGT Colliers de Perles are shown on one layer (on the left hand side) and on two layers with hydrogen bonds (on the right hand side). (A) The IMGT Collier de Perles of a V-DOMAIN is based on the IMGT unique numbering for V-DOMAIN and V-LIKE-DOMAIN [16]. The CDR-IMGT are limited by amino acids shown in squares, which belong to the neighbouring FR-IMGT. The CDR3-IMGT extends from position 105 to position 117. CDR-IMGT regions are colored as follows on the IMGT site: CDR1-IMGT (blue), CDR2-IMGT (bright green), CDR3-IMGT (dark green) and hydrogen bonds are shown as green lines. (B) The IMGT Collier de Perles of a C-DOMAIN is based on the IMGT unique numbering for C-DOMAIN and C-LIKE-DOMAIN [17]. Amino acids are shown in the one-letter abbreviation. Arrows indicate the direction of the beta strands that form the two beta sheets of the immunoglobulin fold [3, 4]. Hatched circles correspond to missing positions according to the IMGT unique numbering [16, 17]. In the IMGT Collier de Perles on the IMGT Web site  hydrophobic amino acids (hydropathy index with positive value) and Tryptophan (W) found at a given position in more than 50 % of analysed IG and TR sequences are shown in blue, and all Proline (P) are shown in yellow.

**Figure 3:**
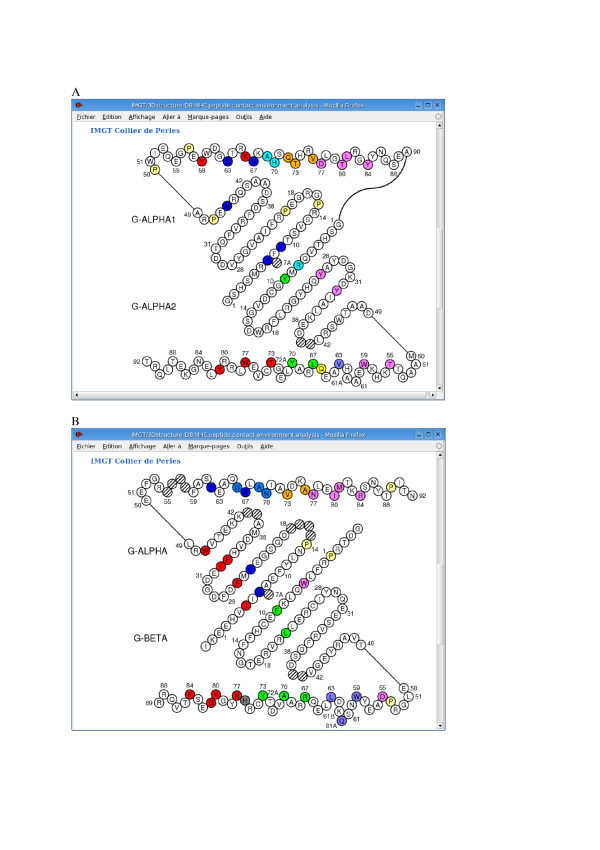
IMGT Colliers de Perles of the two G-DOMAINs of MHC class I (A) and of MHC class II (B) proteins (codes PDB 1bd2 and 1aqd, respectively, in IMGT/3Dstructure-DB [9]). The IMGT Collier de Perles of a G-DOMAIN is based on the IMGT unique numbering for G-DOMAIN and G-LIKE-DOMAIN [18]. (A) The two MHC-I G-DOMAINs, G-ALPHA1 (top) and G-ALPHA2 (bottom), form the groove of the MHC class I chain (I-ALPHA). (B) The two MHC-II G-DOMAINs, G-ALPHA (top) of the MHC class II alpha chain (II-ALPHA) and G-BETA (bottom) of the MHC class II beta chain (II-BETA), form the groove of the MHC class II protein [36]. Amino acids are shown in the one-letter abbreviation. Hatched circles correspond to missing positions according to the IMGT unique numbering [18]. Positions in colour correspond to the IMGT contact sites provided, for each peptide/MHC 3D structure, in IMGT/3Dstructure-DB [36].

Structural and functional domains of the IG and TR chains comprise the variable domain or V-DOMAIN (9-strand beta-sandwich) which corresponds to the V-J-REGION or V-D-J-REGION and is encoded by two or three genes [3,4], the constant domain or C-DOMAIN (7-strand beta-sandwich), and, for the MHC chains, the groove domain or G-DOMAIN (4 beta-strand and one alpha-helix). A uniform numbering system for IG and TR V-DOMAINs of all vertebrate species has been established to facilitate sequence comparison and cross-referencing between experiments from different laboratories whatever the antigen receptor (IG or TR), the chain type, or the species [14-16]. In the IMGT unique numbering, conserved amino acids from frameworks always have the same number whatever the IG or TR variable sequence, and whatever the species they come from. As examples: Cysteine 23 (in FR1-IMGT), Tryptophan 41 (in FR2-IMGT), hydrophobic amino acid 89 and Cysteine 104 (in FR3-IMGT) (Figure 2). This numbering has been applied with success to all the sequences belonging to the V-set of the IgSF [20], including non-rearranging sequences in vertebrates (human CD4, Xenopus CTXg1, etc.) and in invertebrates (drosophila amalgam, drosophila fasciclin II, etc.) [15,16,21]. The IMGT unique numbering, initially defined for the V-DOMAINs of the IG and TR and for the V-LIKE-DOMAINs of IgSF proteins other than IG and TR, has been extended to the C-DOMAINs of the IG and TR (Figure 2B), and to the C-LIKE-DOMAINs of IgSF proteins other than IG and TR [17]. An IMGT unique numbering has also been implemented for the groove domain (G-DOMAIN) of the MHC class I and II chains (Figure 3), and for the G-LIKE-DOMAINs of MhcSF proteins other than MHC [18].

#### IMGT/3Dstructure-DB, the IMGT 3D structure database

IMGT/3Dstructure-DB is the IMGT 3D structure database, created by LIGM, and on the Web since November 2001 [9]. In August 2005, IMGT/3Dstructure-DB contained 946 atomic coordinate files. IMGT/3Dstructure-DB comprises IG, TR, MHC and RPI with known 3D structures [9,36,37]. Coordinate files extracted from the Protein Data Bank (PDB) [38] are renumbered according to the standardized IMGT unique numbering [16-18]. The IMGT/3Dstructure-DB card provides, on-line, the complete information for each IMGT/3Dstructure-DB entry. The IMGT/3Dstructure-DB card shows a summary table and a menu that gives access to five sections: "Chain details", "Contact analysis", "Visualization with Jmol", "Renumbered file" and "References and links". The "Chain details" section provides chain description, IMGT gene and allele names, IMGT chain and domain labels, domain delimitations, amino acid positions according to the IMGT unique numbering, IMGT Colliers de Perles [16-19]. The "Contact analysis" section provides contact types and categories between domains (in *IMGT/3Dstructure-DB Domain contacts*) and atom contacts at the residue and position level (in *IMGT/3Dstructure-DB Residue@Position contacts*) [37]. (IMGT/3Dstructure-DB Documentation). The "Renumbered file" section downloadable provides renumbered IMGT/3Dstructure-DB flat files.

#### IMGT/StructuralQuery tool

The IMGT/StructuralQuery tool [9] analyses the interactions of the residues of the antigen receptors IG and TR, MHC, RPI, antigens and ligands. The contacts are described per domain (intra- and inter-domain contacts) and annotated in term of IMGT labels (chains, domain), positions (IMGT unique numbering), backbone or side-chain implication [37]. IMGT/StructuralQuery allows to retrieve the IMGT/3Dstructure-DB entries, based on specific structural characteristics: phi and psi angles, accessible surface area (ASA), amino acid type, distance in angstrom between amino acids, CDR-IMGT lengths.

#### IMGT structure Web resources

The IMGT stucture Web resources are compiled in the IMGT Repertoire "2D and 3D structures" section which includes 2D representations or IMGT Colliers de Perles [16-19], 3D representations, FR-IMGT and CDR-IMGT lengths [16], amino acid chemical characteristics profiles [31], etc. In order to appropriately analyse the amino acid resemblances and differences between IG, TR, MHC and RPI chains, eleven IMGT classes were defined for the 'chemical characteristics' amino acid properties and used to set up IMGT Colliers de Perles reference profiles [31]. The IMGT Colliers de Perles reference profiles allow to easily compare amino acid properties at each position whatever the domain, the chain, the receptor or the species. The IG and TR variable and constant domains represent a privileged situation for the analysis of amino acid properties in relation with 3D structures, by the conservation of their 3D structure despite divergent amino acid sequences, and by the considerable amount of genomic (IMGT Repertoire), structural (IMGT/3Dstructure-DB) and functional data available. These data are not only useful to study mutations and allele polymorphisms, but are also needed to establish correlations between amino acids in the protein sequences and 3D structures and to determine amino acids potentially involved in the immunogenicity.

## Conclusion

In order to allow any IMGT component to be automatically queried and to achieve a higher level of interoperability inside the IMGT information system and with other information systems, our current objectives include the modelling of the three major IMGT biological approaches, genomics, genetics and structural approaches, the analysis of the IMGT components (databases, tools and Web resources) in relation with the concepts, and the development of Web services [2]. They are the first steps towards the implementation of IMGT-Choreography [2], which corresponds to the process of complex immunogenetics knowledge [25] and to the connection of treatments performed by the IMGT component Web services. IMGT-Choreography has for goal to combine and join the IMGT database queries and analysis tools. In order to keep only significant approaches, a rigorous analysis of the scientific standards [3,4], of the biologist requests and of the clinician needs [39-42] has been undertaken in the three main biological approaches: genomics, genetics and structural approaches. The design of IMGT-Choreography and the creation of dynamic interactions between the IMGT databases and tools, using the Web services and IMGT-ML, represent novel and major developments of IMGT, the international reference in immunogenetics and immunoinformatics. IMGT-Choreography enhances the dynamic interactions between the IMGT components to answer complex biological and clinical requests.

Since July 1995, IMGT has been available on the Web at . IMGT has an exceptional response with more than 140,000 requests a month. The information is of much value to clinicians and biological scientists in general. IMGT databases, tools and Web resources are extensively queried and used by scientists from both academic and industrial laboratories, from very diverse research domains: (i) fundamental and medical research (repertoire analysis of the IG antibody sites and of the TR recognition sites in normal and pathological situations such as autoimmune diseases, infectious diseases, AIDS, leukemias, lymphomas, myelomas), (ii) veterinary research (IG and TR repertoires in farm and wild life species), (iii) genome diversity and genome evolution studies of the adaptive immune responses, (iv) structural evolution of the IgSF and MhcSF proteins, (v) biotechnology related to antibody engineering (single chain Fragment variable (scFv), phage displays, combinatorial libraries, chimeric, humanized and human antibodies), (vi) diagnostics (clonalities, detection and follow up of residual diseases) and (vii) therapeutical approaches (grafts, immunotherapy, vaccinology).

## Citing IMGT

If you use IMGT databases, tools and/or Web resources, please cite [1] and this paper as references, and quote the IMGT Home page URL address, .
